# A Unique Primer with an Inosine Chain at the 5′-Terminus Improves the Reliability of SNP Analysis Using the PCR-Amplified Product Length Polymorphism Method

**DOI:** 10.1371/journal.pone.0136995

**Published:** 2015-09-18

**Authors:** Hideki Shojo, Mayumi Tanaka, Ryohei Takahashi, Tsuneo Kakuda, Noboru Adachi

**Affiliations:** Department of Legal Medicine, Interdisciplinary Graduate School of Medicine and Engineering, University of Yamanashi, 1110 Shimokato, Yamanashi, 409–3898, Japan; Oklahoma State University, UNITED STATES

## Abstract

Polymerase chain reaction-amplified product length polymorphism (PCR-APLP) is one of the most convenient and reliable methods for single nucleotide polymorphism (SNP) analysis. This method is based on PCR, but uses allele-specific primers containing SNP sites at the 3′-terminus of each primer. To use this method at least two allele-specific primers and one “counter-primer”, which serves as a common forward or reverse primer of the allele-specific primers, are required. The allele-specific primers have SNP sites at the 3′-terminus, and another primer should have a few non-complementary flaps at the 5′-terminus to detect SNPs by determining the difference of amplicon length by PCR and subsequent electrophoresis. A major disadvantage of the addition of a non-complementary flap is the non-specific annealing of the primer with non-complementary flaps. However, a design principle for avoiding this undesired annealing has not been fully established, therefore, it is often difficult to design effective APLP primers. Here, we report allele-specific primers with an inosine chain at the 5′-terminus for PCR-APLP analysis. This unique design improves the competitiveness of allele-specific primers and the reliability of SNP analysis when using the PCR-APLP method.

## Introduction

**Fig 1:**
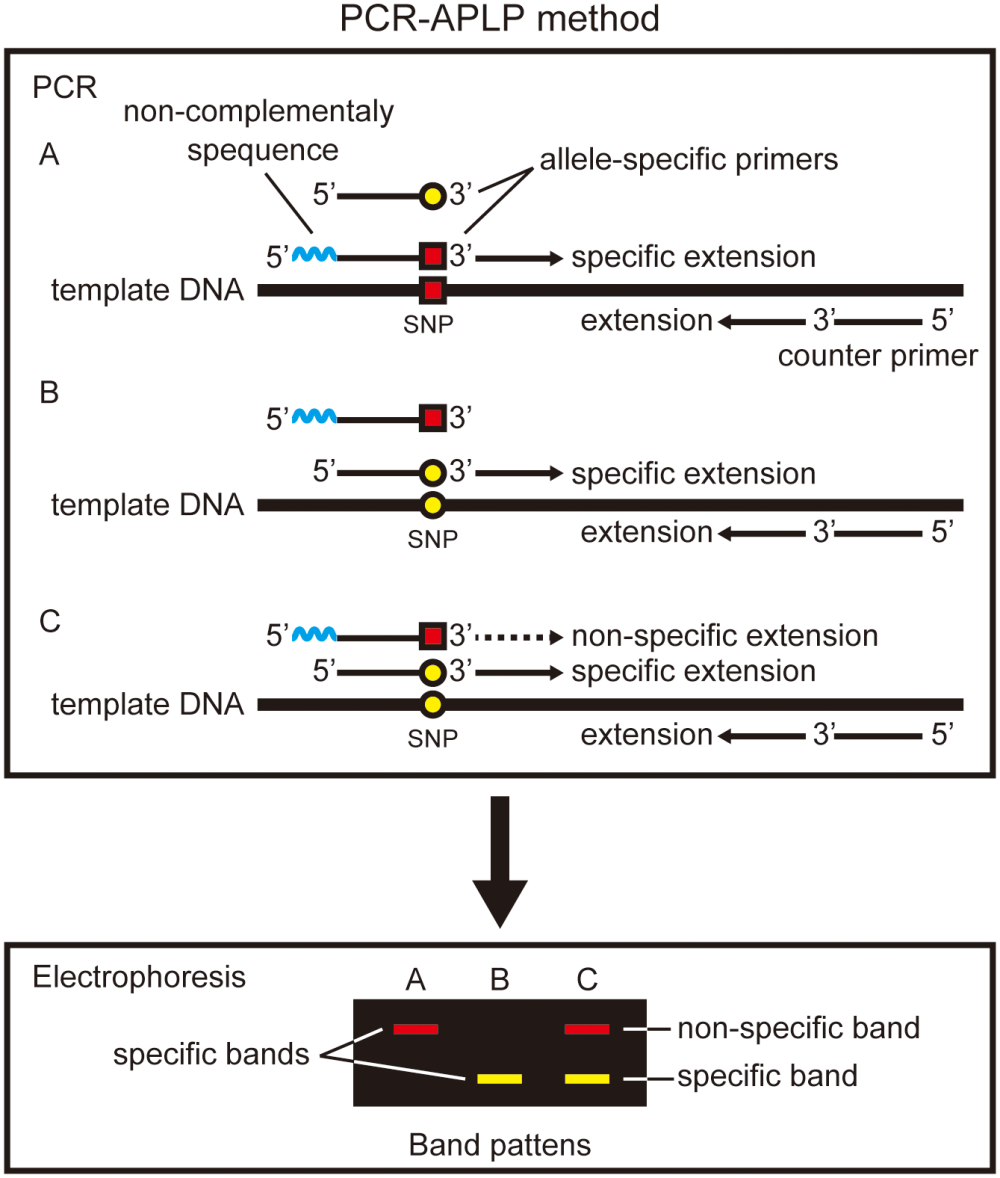
PCR-APLP method for SNP analysis. (A) and (B) Desired extension by specific hybridization of the competitive primer to the SNP site. (C) Unexpected extension by non-specific hybridization of the competitive primer to the SNP site and its electrophoretic banding pattern.

The polymerase chain reaction-amplified product length polymorphism (PCR-APLP) method has been successfully applied for genotyping human blood groups ABO [1, 2] and the haplogrouping of modern and ancient mitochondrial DNA (mtDNA) [3–6], among other applications [7, 8]. This method is based on PCR, but uses allele-specific primers containing SNP sites at the 3′-terminus of each primer. In this method, at least two allele-specific primers and one “counter-primer”, which serves as a common forward or reverse primer of the allele-specific primer set, are required (Fig 1). Each allele-specific primer has SNP sites at the 3′-terminus, and non-complementary bases are added to the 5′-terminus of one primers to allow the detection of SNPs by determining the difference of amplicon length by PCR and subsequent electrophoresis.

The protruding nucleotide fragment at the 5′-terminus of the primer is termed an extension, overhang, flap, or tail, and is used to increase the size of the PCR product, improve the efficiency of the PCR reaction [9, 10, 11, 12] and enhance the quality of sequencing [13]. Primers with a short 5′ A/T-rich flap are reported to increase the fluorescent signal of real-time PCR, and this improvement is particularly significant for sequences that are difficult to amplify, such as bisulfite-treated DNA or highly variable sequences [9, 10]. Afonina et al. reported that the optimal size of the 5′ flap was 12 bp, and adding longer or shorter 5′ flaps had no significant impact on qPCR fluorescence levels [10]. In addition, they demonstrated that G/C-rich flaps were less useful than A/T-rich flaps.

Alternatively, Wei and Clover reported optimum target amplification efficiency using nepovirus primers targeting subgroups A and B using a forward primer with a flap, and a flap-less reverse primer [9]. Although there is no consensus on whether it is better to add flaps to both primers or only one primer, A/T-rich flaps have been used for highly sensitive PCR reactions. Furthermore, Arif and Ochoa-Corona reported that not all primers with 5′ flaps improved the sensitivity of PCR, because the optimal sequence of the 5′ flap varies according to the original primer sequence, and can alter the melting temperature (Tm) value of that primer significantly [11]. Therefore, when designing primers with 5′ flaps, the optimal thermodynamics of the specific primer must be fully understood to improve the sensitivity and specificity of PCR.

For the PCR-APLP method, a major disadvantage of the addition of a 5′ flap is the increase in Tm of one of the competing primers, which leads to undesired annealing to the non-complementary template. The current study investigated the addition of bases to the 5′-terminus of allele-specific primers to improve their competiveness in the PCR-APLP method. Inosine is a ‘universal base’ or a ‘degenerate base’ because of its ability to pair indiscriminately with each of the four standard nucleotide bases, adenine (A), cytosine (C), guanine (G), and thymine (T), via two hydrogen bonds [14–17]. Inosine is therefore commonly used in degenerate primers for the determination of unknown mRNA sequences [18–21].

Inosine binds most stably to cytosine and then in the order of thermodynamic stability: inosine (I)-C > I-A > I-T ≈ I-G [16]. The normal DNA duplexes (G-C, C-G, T-A and A-T) are significantly more stable than those containing inosine, with the exception of C-I and I-C, which are close in stability to A-T [22]. Therefore, the influence of a 5′ flap containing inosine on increased Tm is considered to be similar to that of A/T-rich flaps, and the thermodynamics of primers containing an inosine flap is expected to be influenced less by the sequence of PCR templates than those with 5′ flaps containing ordinary bases. This study reports the design of novel primers with a short inosine extension added to the 5′-terminus to improve their competitiveness to the template DNA resulting in the enhanced reliability of SNP analysis using the PCR-APLP method.

## Materials and Methods

### DNA samples

To obtain DNA, intraoral epithelial cells were collected from two healthy Japanese adults whose mtDNA haplogroups (F and M7a) were inferred from the sequence data of hypervariable region 1 of their mtDNA. The samples were newly collected with a Forensic Swab (Sarstedt Inc., Nümbrecht, Germany). DNA extraction was performed using a MonoFas^®^ Intraoral epithelial cells genome DNA extraction kit VIII (GL Science Inc., Tokyo, Japan), according to the manufacturer’s protocol. The quantity and purity of the DNA was evaluated by optical density (OD)_260_ and OD_260/280_ measurements using a spectrophotometer (NanoDrop 1000; Thermo Fisher Scientific Inc., Waltham, MA, USA). Their mtDNA haplogroups were confirmed to be F and M7a by combining data from a previously established APLP-method and direct sequencing of hypervariable region 1 [5, 6, 23]. This study was approved by the Ethics Committee of the University of Yamanashi.

In addition to these modern samples, we also used ancient DNA samples whose mtDNA haplogroups (D4a, B4a, F1b, N9b, and A) were previously established [5, 23] in experiments for multiplex SNPs analyses using allele-specific primers.

### Binding property of inosine in a PCR mixture, and the electrophoretic mobility of amplicons generated by using inosine flap primers

To assess the binding properties of inosine in a PCR mixture, PCR was performed using a Qiagen Multiplex PCR Master Mix (Qiagen, Hilden, Germany) with the newly designed primers shown in Table 1. A segment of mtDNA (nucleotide position (np) 985–1046, relative to the revised Cambridge reference sequence (rCRS) [24]) was amplified.

**Table 1:** Primers used to assess the binding properties of inosine and the electrophoretic mobility of the resulting amplicon.

Primer name	Sequence	Amplicon size (bp)
L984	TCC CCA ATA AAG CTA AAA CTC ACC	110 + n
Inosine-H1047	(I×n)—GTT TGG GTC TTA GCT ATT GTG TGT	

‘I’ indicates inosine and ‘n’ denotes the number of inosines added to the 5′-terminus, which varied from 0 to 10, or 20.

Amplifications were carried out in a total reaction volume of 10 μL, containing 1 ng of crude DNA, which included genomic DNA and mtDNA with known haplogroups F (a branch of macrohaplogroup N), 0.2 μM of each primer, in 1× Qiagen Multiplex PCR Master Mix (Qiagen). PCR amplification conditions were as follows: incubation at 95°C for 15 min, 36 cycles at 94°C for 30 s, and 64°C for 90 s, followed by a final extension at 72°C for 3 min. Following PCR, the products were subjected to direct sequencing using the L984 primer and the BigDye Terminators v1.1 Cycle Sequencing Kit (Applied Biosystems, Foster City, CA, USA) in a 310 DNA Sequencer (Applied Biosystems) loaded with Ridom TraceEdit software (Ridom, Würzburg, Germany).

To assess the electrophoretic mobility of the amplicons generated using inosine-extended primers, a 2 μL aliquot of the amplicons amplified by the primers in Table 1 were electrophoresed in a 6.5 cm native polyacrylamide gel (10% T, 5% C) containing 1× Tris Borate EDTA (TBE) buffer with running buffer (1× TBE). The number of inosines (n) varied from 0 to 10, or 20. Amplicons were detected fluorographically after staining with SYBR green I (Bio-Rad Laboratories, Hercules, CA, USA).

The result of each experiment was confirmed by three independent assays.

### Thermodynamics of allele-specific primers

To confirm the thermodynamics of allele-specific primers, we compared them with inosine flap primers and primers with ordinary non-complementary flaps by using newly designed N and F primer sets (Table 2). Because the primers used for PCR-APLP have an SNP site at the 3′-terminus, its design is strictly bound by the sequence of the template. For this reason, it is difficult for any software to design PCR-APLP primers. Therefore, we designed the primers manually.

**Table 2:** Primers used for evaluating the effectiveness of inosine in improving competitiveness.

Haplogroups	RCR reaction mixture	Primer name	Sequence	Concentration (μM)
B	Mix 1 and 2	9 bp F	GCC CAT CGT CCT AGA ATT AAT TC	0.2
	Mix 1 and 2	9 bp R	TGC TAA GTT AGC TTT ACA GTG GG	0.2
N	Mix 1	10873T with I	III AGC CTA ATT ATT AGC ATC ATC CC***T***	0.2
	Mix 2	10873T	GTG AGC CTA ATT ATT AGC ATC ATC CC***T***	0.2
	Mix 1 and 2	10873C	GCC TAA TTA TTA GCA TCA TCC C***C***	0.2
	Mix 1 and 2	10873R	GGG GGT CGG AGG AAA AGG T	0.2
R	Mix 1	12705T with I	III CAT TAA TCA GTT CTT CAA ATA TCT ACT CAT ***T***	0.2
	Mix 2	12705T	TTT CAT TAA TCA GTT CTT CAA ATA TCT ACT CAT ***T***	0.2
	Mix 1 and 2	12705C	ATT AAT CAG TTC TTC AAA TAT CTA CTC AT***C***	0.2
	Mix 1 and 2	12705R	AGT TGG AAT AGG TTG TTA GCG GTA A	0.2
F	Mix 1	6392T with I	III CTC TAT CTT AGG GGC CAT CAA ***T***	0.1
	Mix 2	6392T	GAG CTC TAT CTT AGG GGC CAT CAA ***T***	0.1
	Mix 1 and 2	6392C	TCT ATC TTA GGG GCC ATC AA***C***	0.2
	Mix 1 and 2	6392R	GGT ATT GGG TTA TGG CAG GG	0.2
N9	Mix 1	5417A with I	IIII GGG TGG GTT TTG TAT GTT CAA A***T***	0.2
	Mix 2	5417A	TTAC GGG TGG GTT TTG TAT GTT CAA A***T***	0.2
	Mix 1 and 2	5417G	GGG TGG GTT TTG TAT GTT CAA A***C***	0.2
	Mix 1 and 2	5417F	TAC TCC CCA TAT CTA ACA ACG TAA	0.2
A	Mix 1	1736A with I	I ACC TTA GCC AAA CCA TTT ACC CA***A***	0.05
	Mix 2	1736A	T ACC TTA GCC AAA CCA TTT ACC CA***A***	0.05
	Mix 1 and 2	1736G	TTA GCC AAA CCA TTT ACC CA***G***	0.2
	Mix 1 and 2	1736R	GGT TTC AAT TTC TAT CGC CTA TA	0.2

MtDNA haplogroups selected here are the major branches of macrohaplogroup N in East Asia. Underlined letters indicate non-complementary bases. SNP sites are indicated by bold italic type. In Mix 1, primers with inosine flaps and their competing primers, as well as their counter primers, were used. In Mix 2, primers with ordinary non-complementary bases and their competing primers, as well as their counter primers were used.

The N primer set discriminates between macrohaplogroup N and non-N, and the F primer set discriminates between haplogroup F and other haplogroups. One ng of crude DNA, which included genomic DNA and mtDNA with known haplogroups F (a branch of macrohaplogroup N) and M7a (a branch of macrohaplogroup M), were used as template DNA.

PCR reactions were carried out using various annealing temperatures from 60°C to 70°C. Briefly, PCR amplification conditions were as follows: 95°C for 15 min, 36 cycles at 94°C for 30 s and 60–70°C for 90 s, with a final extension at 72°C for 3 min.

The result of the experiment was confirmed by three independent assays.

### Competitiveness and sensitivity of allele-specific primers

To compare the competitiveness and sensitivity between inosine flap primers and primers with ordinary non-complementary flaps, various amounts (1.0 × 10^−9^–0.1 × 10^−19^ g) of mtDNA templates were amplified using N and F primers sets (Table 2). MtDNA templates that contained haplogroup defining SNP sites of haplogroups N and F (np. 10873 and 9362, respectively) were generated as described below.

One ng of crude DNA solution, which included genomic DNA and mtDNA with known haplogroups (F and M7a), were amplified using primer sets N check (L10845: CAC ATA ATT TGA ATC AAC ACA ACC AC and H10951: AGT CAG GTA GTT AGT ATT AGG AGG) and F check (L6350: CGT AGA CCT AAC CAT CTT CTC C and H6458: GCT GTG ATT AGG ACG GAT CAG). PCR conditions were as follows: incubation at 95°C for 15 min, 36 cycles at 94°C for 30 s, and 64°C for 90 s, followed by a final extension at 72°C for 3 min.

PCR products obtained using these primer sets were electrophoresed in a 2% agarose gel containing 1× TAE (Tris Acetate EDTA) buffer with running buffer (1× TAE). Amplicons were detected fluorographically after staining with SYBR green І (Bio-Rad Laboratories). These amplicons were extracted and purified from agarose gel using a MonoFas^®^ DNA purification Kit Ι (GL Science Inc.), according to the manufacturer’s protocol. Amplicons that stemmed from haplogroup F and M7a mtDNAs were mixed equally, diluted with sterilized water from 1.0 × 10^−9^ g/μl to 0.1 × 10^−19^ g/μl, and used as mtDNA templates.

PCR conditions were as follows: 95°C for 15 min, 36 cycles at 94°C for 30 s and 64°C for 90 s, with a final extension at 72°C for 3 min.

The result of the experiment was confirmed by three independent assays.

### Multiplex SNPs analyses by using allele-specific primers

To assess whether the addition of inosine to the 5′-terminus of one of the competing APLP primers improved competitiveness compared to that of ordinary non-complementary flap, the polymorphisms that define major branches of macrohaplogroup N mtDNAs in East Asia were examined by multiplex PCR-APLP using the newly designed primer sets listed in Table 2. In the APLP primers, non-complementary bases were added to the 5′-terminus of one of the competing primers to ensure a difference in the length of amplicons. In general, a difference of about 4 bp is required to separate the amplicons on a gel. Ordinary non-complementary bases at the 5′-terminus achieve complete non-complementarity to the template. Haplogroup B is defined by a 9 bp deletion in the non-coding cytochrome oxidase II/tRNA^Lys^ intergenic region; therefore, the primers detecting this haplogroup do not contain flaps.

MtDNAs with known haplogroups (D4a, B4a, F1b, N9b, and A) were used as DNA templates. Haplogroup D4a is a branch of macrohaplogroup M, which serves as a non-N, R, F, N9, A template. Samples other than D4a belong to macrohaplogroup N. Haplogroup B4a is a branch of haplogroup R, and has a 9 bp deletion. Haplogroup F1b is a branch of haplogroup R and F. Haplogroup N9b is a branch of N9. Samples whose mtDNA belonged to haplogroup A were assigned haplogroup A in this experiment.

Each reaction was performed in 10 μL volume, containing 1 μL template DNA solution, the optimum concentration of each primer in 1 × Qiagen Multiplex PCR Master Mix (Qiagen). Because we used highly degraded ancient DNA as templates, we could not measure the exact amount of template DNA by spectrophotometer. PCR conditions were as follows: 95°C for 15 min, 36 cycles at 94°C for 30 s and 64°C for 90 s (ramp speed = 2.5°C/s), with a final extension at 72°C for 3 min. For the multiplex reaction, the ramp speed should be fast to prevent non-specific hybridization of primers to template DNA. We recommend a ramp speed at over 2.5°C/s. Electrophoretic conditions and visualizing methods of amplicons were the same as described earlier.

### Measurement of Tm values to template probes of allele-specific primers

**Fig 2:**
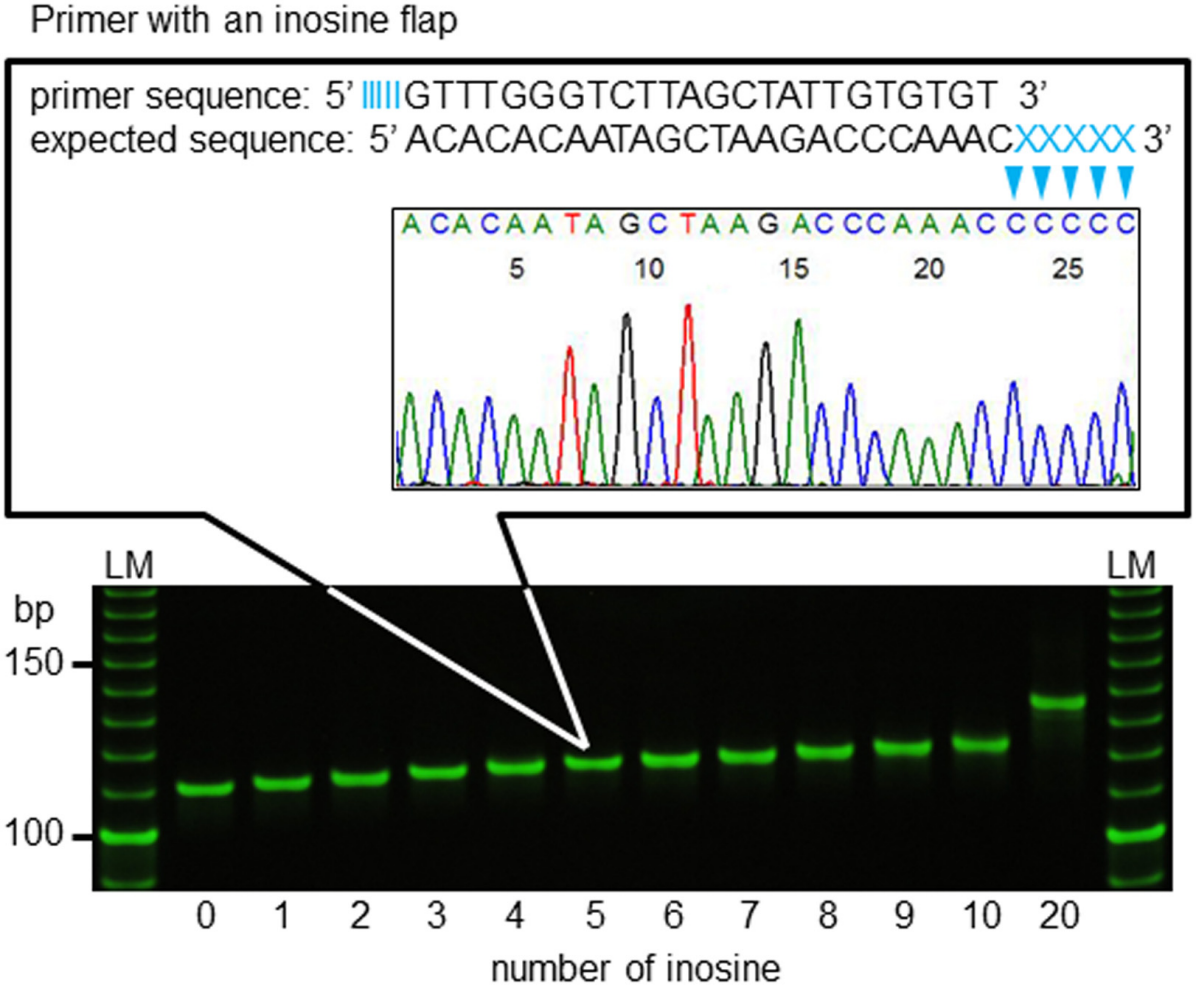
Sequence electropherogram (upper panel) and electrophoretic pattern (lower panel) of the amplicon generated using the inosine flap primer. The sequence data is of the amplicon obtained by the Inosine H1047 primer with five inosines added to the 5′-terminus (Table 1). LM indicates 10 bp ladder marker.

To assess the annealing temperatures of our competitive allele-specific primers described in Table 2, we measured Tm values of inosine flap primers and primers with ordinary non-complementary flaps from the melting curves by using oligonucleotides probes (Table 3). Each oligonucleotide probe, which has the same length as the primer, was synthesized according to the rCRS. As shown in Fig 2, the Tm of inosine flap primers after the second PCR cycle was evaluated by using oligonucleotide probes with the addition of cytosines at the 3′-terminus of the probes, because the 5′-terminal inosine combined mostly with cytosines. Similarly, the Tm of primers with non-complementary flaps after the second PCR cycle was measured by using probes with oligonucleotide flaps that were complementary to the template sequence.

**Table 3:** Tm values of competing primers.

Haplogroup	Primer and probe	Sequence	Tm values (°C)	
			Predicted	Measured
N	10873T with I	III AGC CTA ATT ATT AGC ATC ATC CC***T***		
	Prb-10873T-α-1^st^	***A***GG GAT GAT GCT AAT AAT TAG GCT GTG	62.6	59.0 ± 0.0
	Prb-10873T-β-1^st^	***G***GG GAT GAT GCT AAT AAT TAG GCT GTG	61.2	58.8 ± 0.2
	Prb-10873T-α-IF-2^nd^	***A***GG GAT GAT GCT AAT AAT TAG GCT CCC	64.1	59.5 ± 0.0
	10873T	GTG AGC CTA ATT ATT AGC ATC ATC CC***T***		
	Prb-10873T-α-1^st^	***A***GG GAT GAT GCT AAT AAT TAG GCT GTG	62.6	59.5 ± 0.0
	Prb-10873T-β-1^st^	***G***GG GAT GAT GCT AAT AAT TAG GCT GTG	61.2	58.8 ± 0.2
	Prb-10873T-α-NF-2^nd^	***A***GG GAT GAT GCT AAT AAT TAG GCT CAC	66.5	63.0 ± 0.0
non-N	10873C	GCC TAA TTA TTA GCA TCA TCC C***C***		
	Prb-10873C-α-1^st^ and 2^nd^	***G***GG GAT GAT GCT AAT AAT TAG GC	62.1	59.5 ± 0.0
	Prb-10873C-β-1^st^	***A***GG GAT GAT GCT AAT AAT TAG GC	60.5	58.3 ± 0.2
R	12705T with I	III CAT TAA TCA GTT CTT CAA ATA TCT ACT CAT ***T***		
	Prb-12705T-α-1^st^	***A***AT GAG TAG ATA TTT GAA GAA CTG ATT AAT GTT T	62.9	59.0 ± 0.0
	Prb-12705T-β-1^st^	***G***AT GAG TAG ATA TTT GAA GAA CTG ATT AAT GTT T	62.4	58.5 ± 0.0
	Prb-12705T-α-IF-2^nd^	***A***AT GAG TAG ATA TTT GAA GAA CTG ATT AAT GCC C	64.4	59.7 ± 0.2
	12705T	TTT CAT TAA TCA GTT CTT CAA ATA TCT ACT CAT ***T***		
	Prb-12705T-α-1^st^	***A***AT GAG TAG ATA TTT GAA GAA CTG ATT AAT GTT T	62.7	58.7 ± 0.2
	Prb-12705T-β-1^st^	***G***AT GAG TAG ATA TTT GAA GAA CTG ATT AAT GTT T	62.1	58.5 ± 0.0
	Prb-12705T-α-NF-2^nd^	***A***AT GAG TAG ATA TTT GAA GAA CTG ATT AAT GAA A	64.6	60.0 ± 0.0
non-R	12705C	ATT AAT CAG TTC TTC AAA TAT CTA CTC AT***C***		
	Prb-12705C-α-1^st^ and 2^nd^	***G***AT GAG TAG ATA TTT GAA GAA CTG ATT AAT	61.7	58.5 ± 0.0
	Prb-12705C-β-1^st^	***A***AT GAG TAG ATA TTT GAA GAA CTG ATT AAT	60.9	57.7 ± 0.2
F	6392T with I	III CTC TAT CTT AGG GGC CAT CAA ***T***		
	Prb-6392T-α-1^st^	***A***TT GAT GGC CCC TAA GAT AGA GGA G	64.5	58.5 ± 0.0
	Prb-6392T-β-1^st^	***G***TT GAT GGC CCC TAA GAT AGA GGA G	63.7	58.0 ± 0.0
	Prb-6392T-α-IF-2^nd^	***A***TT GAT GGC CCC TAA GAT AGA GCC C	65.4	59.8 ± 0.2
	6392T	GAG CTC TAT CTT AGG GGC CAT CAA ***T***		
	Prb-6392T-α-1^st^	***A***TT GAT GGC CCC TAA GAT AGA GGA G	64.5	58.8 ± 0.2
	Prb-6392T-β-1^st^	***G***TT GAT GGC CCC TAA GAT AGA GGA G	63.7	58.0 ± 0.0
	Prb-6392T-α-NF-2^nd^	***A***TT GAT GGC CCC TAA GAT AGA GCT C	68.2	63.2 ± 0.2
non-F	6392C	TCT ATC TTA GGG GCC ATC AA***C***		
	Prb-6392C-α-1^st^ and 2^nd^	***G***TT GAT GGC CCC TAA GAT AGA	61.7	58.5 ± 0.0
	Prb-6392C-β-1^st^	***A***TT GAT GGC CCC TAA GAT AGA	60.7	56.3 ± 0.2
N9	5417A with I	IIII GGG TGG GTT TTG TAT GTT CAA A***T***		
	Prb-5417A-α-1^st^	***A***TT TGA ACA TAC AAA ACC CAC CC CAT T	62.8	59.5 ± 0.0
	Prb-5417A -β-1^st^	***G***TT TGA ACA TAC AAA ACC CAC CC CAT T	62.2	59.0 ± 0.0
	Prb-5417A-α-IF-2^nd^	***A***TT TGA ACA TAC AAA ACC CAC CC CCC C	64.0	60.0 ± 0.0
	5417A	TTAC GGG TGG GTT TTG TAT GTT CAA A***T***		
	Prb-5417A-α-1^st^	***A***TT TGA ACA TAC AAA ACC CAC CC CAT T	62.5	59.3 ± 0.2
	Prb-5417A -β-1^st^	***G***TT TGA ACA TAC AAA ACC CAC CC CAT T	61.8	58.5 ± 0.0
	Prb-5417A-α-NF-2^nd^	***A***TT TGA ACA TAC AAA ACC CAC CC GTA A	66.7	62.3 ± 0.2
non-N9	5417G	GGG TGG GTT TTG TAT GTT CAA A***C***		
	Prb-5417G-α-1^st^ and 2^nd^	***G***TT TGA ACA TAC AAA ACC CAC CC	62.6	60.5 ± 0.0
	Prb-5417G-β-1^st^ and 2^nd^	***A***TT TGA ACA TAC AAA ACC CAC CC	61.8	58.5 ± 0.0
A	1736A with I	I ACC TTA GCC AAA CCA TTT ACC CA***A***		
	Prb-1736A-α-1^st^	***T***TG GGT AAA TGG TTT GGC TAA GGT T	66.2	61.5 ± 0.0
	Prb-1736A-β-1^st^	***C***TG GGT AAA TGG TTT GGC TAA GGT T	65.4	61.5 ± 0.0
	Prb-1736A-α-IF-2^nd^	***T***TG GGT AAA TGG TTT GGC TAA GGT C	66.5	61.5 ± 0.0
	1736A	T ACC TTA GCC AAA CCA TTT ACC CA***A***		
	Prb-1736A-α-1^st^	***T***TG GGT AAA TGG TTT GGC TAA GGT T	66.0	62.0 ± 0.0
	Prb-1736A-β-1^st^	***C***TG GGT AAA TGG TTT GGC TAA GGT T	65.2	61.5 ± 0.0
	Prb-1736A-α-NF-2^nd^	***T***TG GGT AAA TGG TTT GGC TAA GGT A	66.3	61.8 ± 0.2
non-A	1736G	TTA GCC AAA CCA TTT ACC CA***G***		
	Prb-1736G-α-1^st^ and 2^nd^	***C***TG GGT AAA TGG TTT GGC TAA	60.8	58.2 ± 0.2
	Prb-1736G-β-1^st^	***T***TG GGT AAA TGG TTT GGC TAA	59.2	56.5 ± 0.0

Prb denotes probe. ‘α’ indicates that the 3′-terminus of the probe is complementary to the target primer, and ‘β’ denotes that the 3′-terminus of the probe is non-complementary to the target primer. 1^st^ indicates that the probe was used to measure the Tm of the target primer at the first PCR cycle, and 2^nd^ denotes that the probe was used to measure the Tm of the target primer in later PCR cycles. Mean values ± SD are shown for triplicate assays.

The result of each experiment was confirmed by three independent assays.

Each reaction was performed in a total reaction volume of 20 μL, containing 100 nM of each probe, 100 nM of each primer, and reagents of the 1× Precision Melt Supermix (Bio-Rad Laboratories Inc.). Each probe and primer was completely dissociated at 95°C for 1 min and gently hybridized at 40°C for 1 min, and then melted with stepwise raising temperature between 40°C to 95°C (ramp speed 0.5°C/30 s: hold for 30 s). The melting curves of each probe and primer were represented by using MiniOpticon real-time PCR system (Bio-Rad Laboratories Inc.). The Tm values are indicated as means ± standard deviation from triplicate asssays.

The data of melting analyses was generated by SYBR alternative dye EvaGreen [Bio-Rad Laboratories Inc.). EvaGreen was reported to be suitable for high-resolution melting analysis as compared to SYBR green [25, 26].

In addition, Tm values of haplogroup F and non-F primers were confirmed by UV melting experiments using a Shimadzu spectrophotometer 1800 with thermal melt system TMSPC-8 (Shimadzu Corp., Kyoto, Japan). These results were similar to those obtained by melting analyses using real-time PCR system (data not shown).

Moreover, Tm values of these primers and probes were predicted by using Visual-OMP software (DNA Software Inc., Ann Arbor, MI, USA), because only this software enables the prediction of Tm values of primers containing inosine. The presented values were calculated under PCR application conditions: 0.05 M monovalent cation, 0.0015 M Mg ^2+^, primers and probes concentrations are shown in Table 2. Other parameters were the same as the default settings (PCR with AmpliTaq).

## Results

### PCR products generated using inosine flap allele-specific primers

Sequence data showed that the 5′-terminal inosine section of the primer combined mainly with cytosines (Fig 2). Amplicon size increased according to the number of inosines added to the 5′-terminus of the Inosine H1047 primer.

### Thermodynamics of allele-specific primers

**Fig 3:**
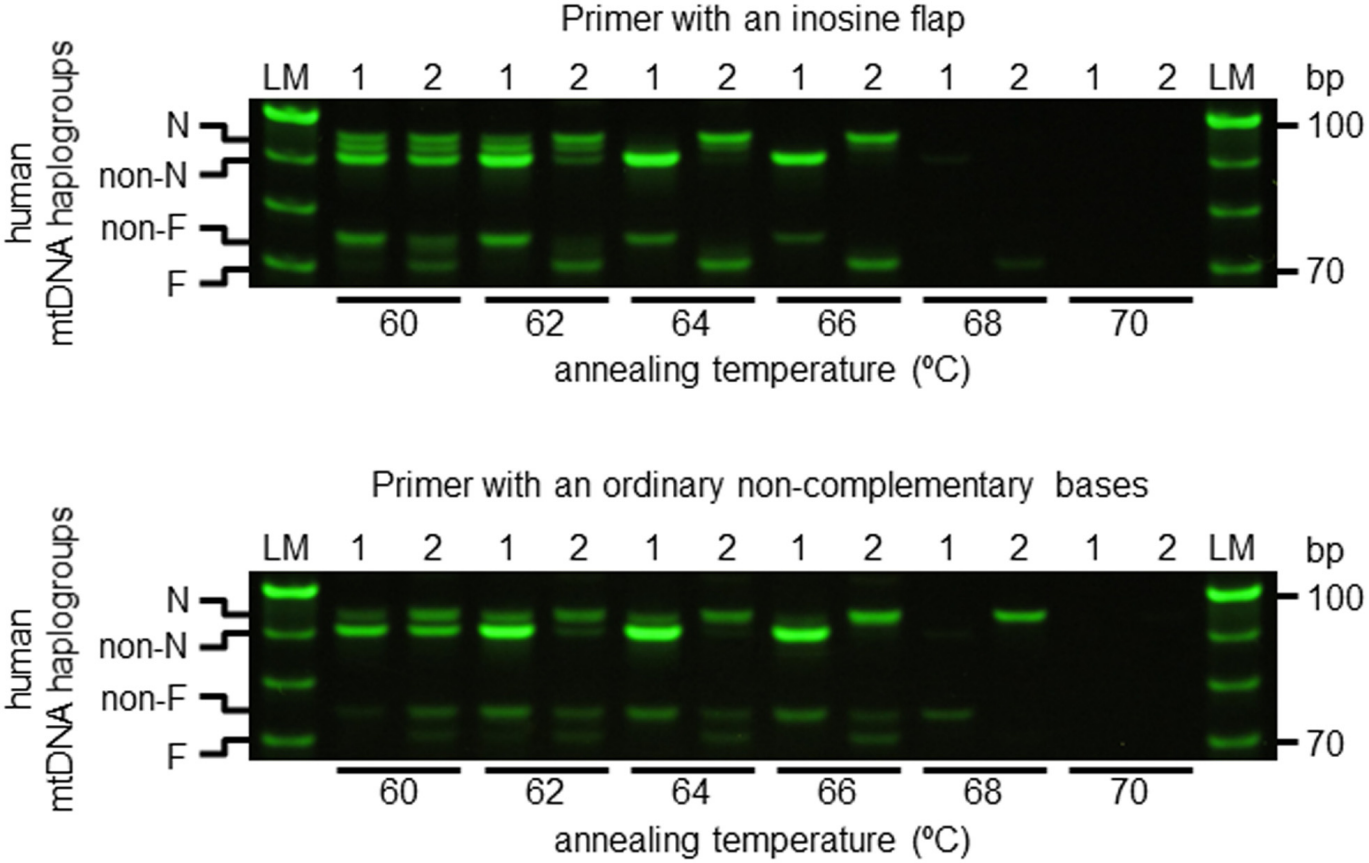
Thermodynamics of allele-specific primers. PCR reactions were carried by using N and F primer sets at various annealing temperatures from 60°C to 70°C. PCR amplification conditions are described in Materials and Methods. Upper panel shows electrophoretogram of the amplicon generated by the inosine flap primer and competitive allele-specific primer. Lower panel shows electrophoretogram of the amplicon generated using the primer with a flap of ordinary non-complemental bases and a competitive allele-specific primer. Sample 1 indicates human mtDNA haplogroup non-N and non-F, and sample 2 indicates human mtDNA haplogroup F. Haplogroup F is a branch of macrohaplogroup N; therefore, this mtDNA is also assigned to haplogroup N. LM indicates 10 bp ladder marker.

Inosine flap primers correctly determined haplogroups N and F at the annealing temperatures of 64–66°C (Fig 3).

Regarding the inosine flap primers, when the annealing temperature was below 62°C, non-specific bands were observed. At higher annealing temperatures over 68°C, all amplicons disappeared (Fig 3). In contrast, for primers with ordinary non-complementary flaps, non-specific bands were observed between 60–66°C, and all bands disappeared over 68°C (Fig 3). Therefore, primers with ordinary non-complementary flaps were not able to correctly identify haplogroups at all annealing temperatures. These results indicated that inosine flap primers anneal more preferentially to template DNA than primers with ordinary non-complementary flaps at annealing temperatures between 64–66°C.

### Competitiveness and sensitivity of allele-specific primers

**Fig 4:**
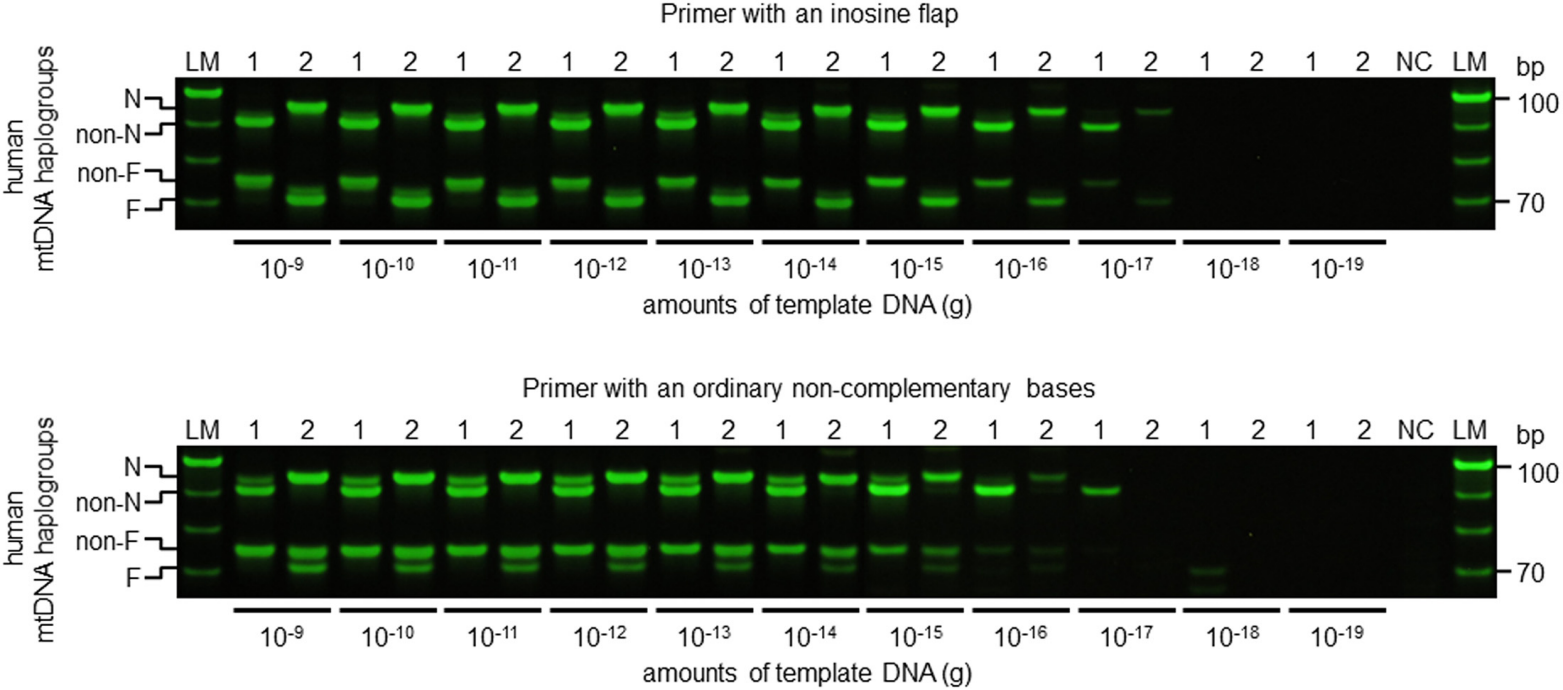
Competitiveness and sensitivity of allele-specific primers. MtDNA templates contained mixtures of haplogroups M7a (non-N and non-F; Sample 1) or F (sample 2) mtDNA. Templates (1.0 × 10^−9^–0.1 × 10^−19^ g) were amplified using N and F primer sets as shown in Materials and Methods. Upper panel shows electrophoretogram of the amplicons generated using the inosine flap primer and the competitive allele-specific primer. Lower panel shows electrophoretogram of the amplicons generated using a primer with a flap of ordinary non-complementary bases and a competitive allele-specific primer. PCR amplification conditions are described in Materials and Methods. NC indicates negative PCR control. LM indicates 10 bp ladder marker.

Inosine flap primers correctly identified haplogroups N and F or non-N and non-F from 1.0 × 10^−17^ g of mixed mtDNA templates, which approximately corresponded to 10 copy numbers by theoretical calculation (Fig 4). By contrast, for primers with non-complementary flaps, non-specific bands were observed at various template amounts (from 1.0 × 10^−9^ to 0.1 × 10^−19^ g, Fig 4). These results showed that inosine flap primers have higher competitiveness and sensitivity to template DNA than those with non-complementary base flaps.

### Multiplex SNPs analyses using allele-specific primers

**Fig 5:**
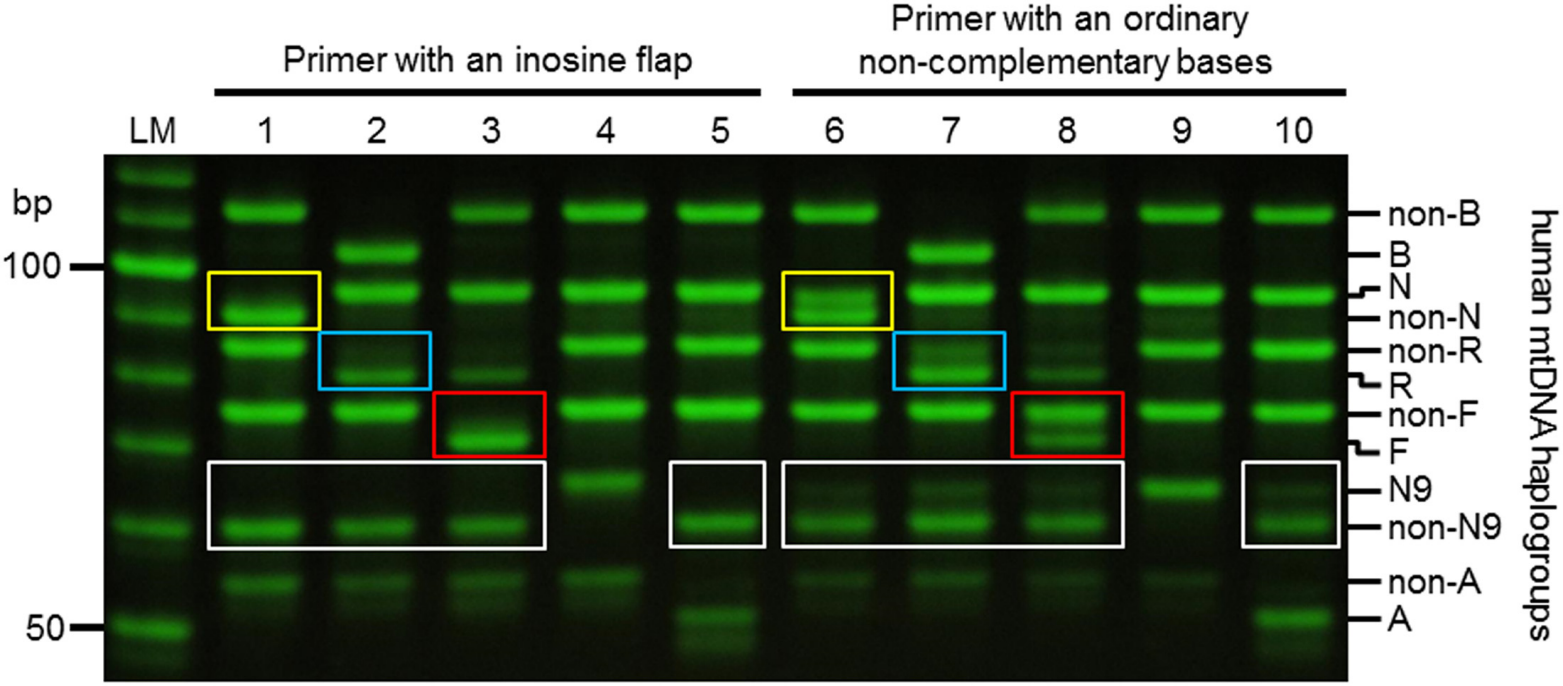
Comparison between PCR-APLP SNP analyses using the inosine flap primer and primer with a flap of ordinary non-complementary bases. Yellow, blue, red, and white open boxes indicate human mtDNA haplogroups N, R, F, and N9, respectively. LM indicates 10 bp ladder marker. Lanes 1 and 6: haplogroup D4a mtDNA; lanes 2 and 7: haplogroup B4a mtDNA; lanes 3 and 8: haplogroup F1b mtDNA; lanes 4 and 9, haplogroup N9b mtDNA; and lanes 5 and 10, haplogroup A mtDNA.

Using ancient DNA samples, we performed multiplex SNP analyses of human mtDNA using inosine flap primers and primers with ordinary non-complementary flaps. It was revealed that inosine flap primers improved competitiveness remarkably compared with primers with ordinary non-complementary flaps, as determined by a decrease of non-specific bands (Fig 5).

Interestingly, it was revealed that the mobility of the amplicons generated by the inosine flap primers and the primers with ordinary non-complementary flap (the amplicons N, non-R, non-F, N9, and non-A) were similar. Therefore, the effect of the inosine flap on the mobility is similar to that of the ordinary non-complementary flap. Moreover, with regards to mobility, the length of the flap is important, whereas the sequence of the flaps, primers and templates are less important.

### Tm values of allele-specific primers

**Fig 6:**
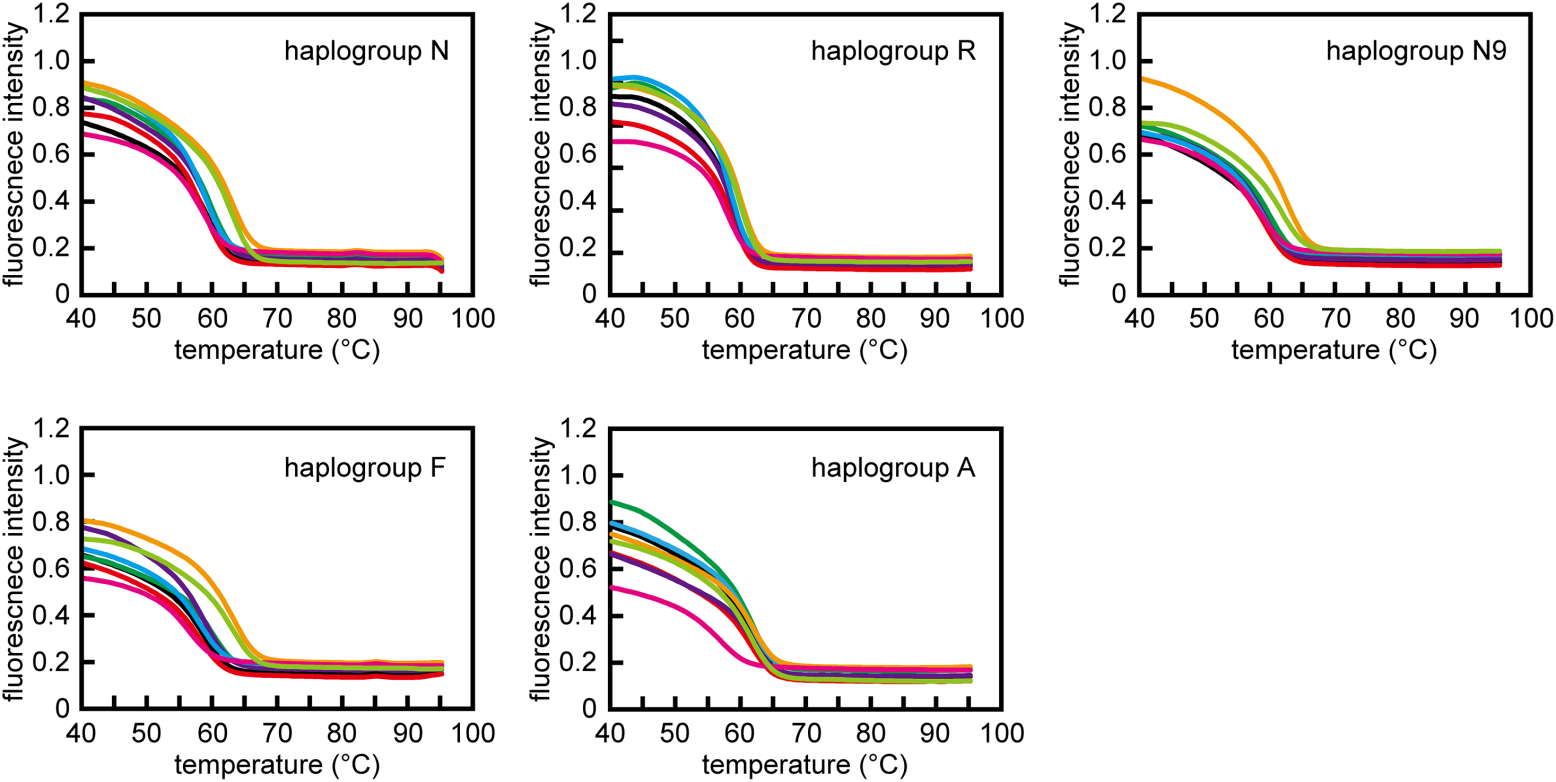
Melting curves of primer-probe pairs of N, R, N9, F and A sets, shown in Table 3. Melting curves of primers are shown as coloured lines as follows: black ‘Primer with inosine (I) and Prb-α-1st’, red ‘Primer with I and Prb-β-1st’, green ‘Primer with I and Prb-α-IF-2nd, purple ‘Primer with a flap of ordinary non-complementary bases and Prb-α-1st’, blue ‘Primer with a flap of ordinary non-complementary bases and Prb-β-1st’, orange ‘Primer with a flap of ordinary non-complementary bases and Prb-α-NF-2nd’, pink ‘Primer without a flap and Prb-α-1st and 2nd’, and yellow-green ‘Primer without a flap and Prb-β-1st and 2nd’. Each Tm value shown in Table 3 was obtained from the melting curves by differentiation. Values are means for triplicate assays.

At the first PCR cycle, Tm values of all ‘primer and Prb-α-1^st^ pairs’ were the same or higher than those of ‘primer and Prb-β-1^st^ pairs’ (Table 3 and Fig 6). This result indicated that, at the first PCR cycle, the competing primers preferably annealed to their correct templates by discriminating the SNPs at the 3′-terminus of the templates regardless of the bases in the flaps.

At later PCR cycles, Tm values of ‘inosine flap primer and Prb-α-IF-2^nd^ pairs’ increased slightly compared with those of primer and Prb-α-1^st^ probe pairs’ (increase of ΔTm was N: 0.5°C, R: 0.7°C, F: 1.3°C, N9: 0.5°C, and A: 0°C, respectively). The Tm values of ‘non-complementary base flap primers and Prb-α-NF-2^nd^ pairs’ increased significantly compared to those of ‘primer and Prb-α-1^st^ pairs’ except for the case of haplogroup A (increase of ΔTm was N: 3.5°C, R: 1.3°C, F: 4.4°C, N9: 3.0°C and A: 0.2°C, respectively).

It should be noted that Tm values of ‘non-complementary base flap primers and Prb-α-NF-2^nd^ pairs’ were obviously higher than those of their competing ‘primer and Prb-α-1^st^ and 2^nd^ pairs’ (difference of ΔTm was N: 3.5°C, R:1.5°C, F:4.7°C, N9:1.8°C and A:3.6°C, respectively). Therefore, non-complementary base flap primer are more prone to undesired annealing to the non-specific template than an inosine flap primer, resulting in generating a non-specific amplicon. These findings might explain why a 5′ flap containing inosine improved the separation of amplicons in PCR-APLP analysis compared with a 5′ flap containing an ordinary non-complementary flap.

Moreover, Tm values of these primers and probes were predicted by using Visual-OMP software. The results showed that the predicted Tm values of primer-probe pairs were about 2–5°C higher than those of the measured values; however, the ΔTm value of each primer-probe pair was similar to that of the measured value (Table 3).

## Discussion

The current study confirmed that the 5′-terminal inosine chain combined mainly with cytosines in a PCR reaction mixture. This is not surprising given that inosine pairs more stably to cytosine compared with T, G, or A [17]. Our result indicates that the counter primer would form a cytosine chain after the second PCR cycle. Moreover, the amplicon size increased according to the number of inosines attached to the 5′-terminus. It is therefore easy to alter the amplicon length by using competitive allele-specific primers with and without inosine chains.

Allele-specific primers used for SNP analysis must be designed to anneal specifically to the segment that includes the target SNPs. Our data indicated that the Tm of all primers with flaps, except for haplogroup A, was higher than that of the competitive primers without flaps in the first PCR cycle (Table 3 and Fig 6). After the second PCR, the Tm of primers with ordinary non-complementary flaps became much higher than that of their competitive primers. Therefore, compared with a competitive allele-specific primer, a primer with an ordinary non-complementary flap can hybridize to a non-complementary template, resulting in the generation of non-specific amplicons by unexpected elongation because of the higher Tm value.

In contrast, as shown in Fig 3, non-specific bands were not observed when using primers with 5′ inosine extension. This is because the Tm value of inosine flap primers remain similar to that in the first PCR cycle, resulting in facilitation of specific annealing of the primers. Therefore, a 5′inosine extension primer markedly improved competitiveness to template DNA compared with a primer with an ordinary non-complementary flap.

However, the thermodynamic properties of the primers to detect haplogroup A were different from those of the other haplogroups. As shown in Table 3, the Tm values of the ‘primer-probe pair 1736A with I and Prb-1736A-β-1^st^’ and ‘primer-probe pair 1736A and Prb-1736A-β-1^st^’ (both = 61.5±0.0°C) were higher than the ‘primer-probe pair 1736G and Prb-1736G-α-1^st^ and 2^nd^’ (58.2±0.2°C). This might explain why the addition of flaps was not as effective in this primer set, and why it is important to control the competitiveness of the primers to template DNA by differences in concentrations of competing primers. Wei and Clover reported optimum target amplification efficiency using a 5′ flap on the forward primer [9]. The Tm values of their forward and reverse primers were 50.4°C and 58.7°C, respectively. The different Tm (8.3°C) between these two primers may render the PCR amplification less efficient. As well as this phenomenon, difference of Tm between competitive primers may have an influence on the competitiveness of allele-specific primers in PCR-APLP.

The normal DNA duplexes (G-C, C-G, T-A and A-T) are significantly more stable than those containing inosine, with the exception of C-I and I-C, which are close in stability to A-T [17, 22]. Primers with A/T-rich flaps have been used to develop new detection/diagnostics methods and to facilitate the efficiency of PCR [9–12]. In APLP primer sets, non-complementary bases are used to change the length of amplicons. In general, a difference of about 4 bp is required to separate amplicons on a gel. Given these findings, primers with short 5′ inosine flaps are expected to improve the efficiency of PCR regardless of the template sequence. In this study, the detection limit of our PCR-APLP method was approximately 10 copies of template DNA. To improve the sensitivity and competitiveness of this reaction, we will investigate the optimal length of the inosine flap.

Recently, various studies have successfully reported the thermodynamic properties of inosine [17]. To allow accurate predictions, the nearest-neighbor parameters of inosine reported here have been added to Visual-OMP software [17, 27]. Although the predicted Tm value of each primer and probe pair was generally higher than the measured values, differences of Tm values between primer and probe pairs (ΔTm) correlated well with those of the measured ΔTm values. Thus, it is now possible to design primers containing inosines with optimized thermodynamic parameters using Visual-OMP software. Furthermore, inosine has been widely applied to DNA microarrays [14, 28–34], and more analytical technologies relying on inosine will likely be developed in the near future.

High-throughput SNP analyses have been applied to various fields [35–37]. These technologies enable the analysis of enormous numbers of SNPs in a large number of samples simultaneously. However, the reliability of these data relies heavily on various statistical algorithms [38–45] and the results must be verified using classical wet-lab methods such as the PCR-APLP method described here. By combining a low-throughput, but more reliable method with high-throughput analyses, will enhance the quality of SNP analyses in various medical and biological fields.

In conclusion, a unique primer design with an inosine extension at the 5′-terminus improved the competitiveness of the primer to template DNA, resulting in an enhanced reliability of SNP analysis when using PCR-APLP. Moreover, this increase in competitiveness using a 5′ inosine flap can be applied to other methods such as microarray analyses, where competitiveness of the primers is critical.
